# The Two Sides of Linguistic Context: Eye-Tracking as a Measure of Semantic Competition in Spoken Word Recognition Among Younger and Older Adults

**DOI:** 10.3389/fnhum.2020.00132

**Published:** 2020-04-09

**Authors:** Nicolai D. Ayasse, Arthur Wingfield

**Affiliations:** Volen National Center for Complex Systems, Brandeis University, Waltham, MA, United States

**Keywords:** eye-tracking, aging, inhibitory control, semantic competition, linguistic context

## Abstract

Studies of spoken word recognition have reliably shown that both younger and older adults’ recognition of acoustically degraded words is facilitated by the presence of a linguistic context. Against this benefit, older adults’ word recognition can be differentially hampered by interference from other words that could also fit the context. These prior studies have primarily used off-line response measures such as the signal-to-noise ratio needed for a target word to be correctly identified. Less clear is the locus of these effects; whether facilitation and interference have their influence primarily during response selection, or whether their effects begin to operate even before a sentence-final target word has been uttered. This question was addressed by tracking 20 younger and 20 older adults’ eye fixations on a visually presented target word that corresponded to the final word of a contextually constraining or neutral sentence, accompanied by a second word on the computer screen that in some cases could also fit the sentence context. Growth curve analysis of the time-course of eye-gaze on a target word showed facilitation and inhibition effects begin to appear even as a spoken sentence is unfolding in time. Consistent with an age-related inhibition deficit, older adults’ word recognition was slowed by the presence of a semantic competitor to a degree not observed for younger adults, with this effect operating early in the recognition process.

## Introduction

Older adults, like young adults, are known to make effective use of linguistic context to facilitate spoken word recognition, especially when the speech signal is degraded by background noise or hearing impairment (Cohen and Faulkner, 1983; Pichora-Fuller et al., 1995; Dubno et al., 2000; Benichov et al., 2012). The reliability of this finding has led to its inclusion in virtually all models of word recognition (see Morton, 1969; Forster, 1981; Marslen-Wilson and Zwitserlood, 1989; Luce and Pisoni, 1998; McClelland et al., 2006; Rönnberg et al., 2019). There are, however, two sides to the influence of a linguistic context on word recognition. Although hearing a word within a linguistic context will facilitate recognition by raising its probability above its initial resting state, the perceptual system must also inhibit a potentially large number of words that might also be activated by the sentence context (Lash et al., 2013; Magnuson et al., 2013).

A dominant position in the literature on cognitive aging is that older adults show a general inhibition deficit (Hasher and Zacks, 1988; Lustig et al., 2007). As such, one would expect semantic competition to retard word recognition to a differentially greater degree for older adults than for younger adults. Extant data support this position in terms of both phonological and semantic competition. For example, older adults require a differentially more favorable signal to noise ratio for recognizing spoken words that have a large number of other words that share their phonology (Sommers, 1996; Sommers and Danielson, 1999). In terms of semantic competition, it has been shown that the amount of word onset information needed for recognition of a word in a sentence context is facilitated by the likelihood of the target word within the sentence, but also adversely affected by the probability distribution of alternative words that might also fit that context. As in the case of phonological competition, the detrimental effect of the semantic competition is differentially greater for older than for younger adults (Lash et al., 2013; Amichetti et al., 2018).

Studies of context effects on word recognition, however, have been typified by two factors that limit interpretation. The first has been a primary focus on the recognition of degraded stimuli, such as using a “gating” paradigm in which the effect of a linguistic context is indexed by the probability of a word being correctly identified as its onset duration is progressively increased (Wingfield et al., 1991; Grosjean, 1996; Lash et al., 2013), or more commonly, by measuring the signal to noise ratio needed for identification of noise-masked words (e.g., Cohen and Faulkner, 1983; Pichora-Fuller et al., 1995; Dubno et al., 2000; Benichov et al., 2012). The second factor is that such studies have primarily relied on off-line measures in the form of participant responses given some time after the degraded stimulus has been presented.

Reliance on off-line responses in studies of context effects on word recognition makes it difficult to determine whether a postulated age-related inhibition deficit appears in an early-stage suppression of non-congruent stimuli, or whether the difficulty occurs in post-perceptual editing of inappropriate responses (Getzmann et al., 2015a,b). For this purpose, one needs an on-line measure that will reveal how the factors of stimulus expectancy and response competition affect the actual moment a target word has been recognized. One solution is to use an adaptation of the “visual world” paradigm.

A common procedure using the “visual world” paradigm is the measurement of the speed of eye fixation on a named target, typically represented by a picture of the object presented along with “distractor” pictures on a computer screen (see reviews in Tanenhaus et al., 2000; Huettig et al., 2011; Van Engen and McLaughlin, 2018). It is presumed that the increased likelihood of eye fixations on a pictured object corresponds to, and is the result of, increased activation of a mental representation of that object or word (e.g., Altmann and Kamide, 2007). A variation of this paradigm using printed words has also been validated (Huettig and McQueen, 2007; McQueen and Viebahn, 2007; Salverda and Tanenhaus, 2010). In both cases, studies with young adults have shown that, as one hears a target word being uttered, participants’ eye fixations tend to dwell increasingly more time on the named object, and correspondingly less time on non-target items also present on the computer screen.

There are two important features of this method. The first is that an individual’s eye-gaze to a pictured object or written word can be closely time-locked to its reference in a spoken utterance (Cooper, 1974). The advantage of this technique lies in the rapidity of an eye fixation on a visual target. This has allowed investigators to elucidate, for example, the relative importance to word recognition of word-onsets vs. word-endings (Allopenna et al., 1998; Magnuson et al., 2007; Ben-David et al., 2011), and effects of working memory capacity on fixation times to a named target (Hadar et al., 2016; Nitsan et al., 2019).

The second feature of this method is that, unlike motor responses such as a key-press or an overt verbal response, the speed of saccadic eye movements show minimal age differences (Pratt et al., 2006). This offers a measure of when recognition of a word has occurred that does not inherently put older adults at a disadvantage (Ayasse et al., 2017).

There have been several studies showing the value of eye-tracking as an on-line measure of the time-course of spoken word recognition in young adults (e.g., Crain and Steedman, 1985; Chambers et al., 2002; Spivey et al., 2002; Dahan and Tanenhaus, 2004; Huettig and McQueen, 2007; Magnuson et al., 2008; Nozari et al., 2016). Although fewer studies have used the visual world paradigm with older adults, there is evidence from eye-gaze studies that the speed of word recognition by older adults is differentially slowed by phonological competition (Ben-David et al., 2011; Revill and Spieler, 2012) and by competing speech (Helfer and Staub, 2014).

Although arguments for an age-related inhibition deficit have had prominence in the field of cognitive aging, there has been less agreement about the locus of older adults’ susceptibility to inference (see Burke and College, 1997; Zacks and Hasher, 1997; Burke and Osborne, 2007). This debate has centered on uncertainty as to whether a postulated age-related inhibition deficit appears in early-stage processing, or whether the difficulty occurs in post-perceptual processing (Getzmann et al., 2015a,b).

In the present experiment, we employed a version of the visual world paradigm to determine whether evidence of an age-related inhibitory deficit appears in an on-line measure of when a word has been recognized. Should this be the case it would be reflective of an inhibition effect in early-stage recognition processing as indexed by differences in the time to an immediate eye-gaze on a semantically constrained target word with and without the presence of a semantic competitor.

## Materials and Methods

### Participants

Participants were 20 younger adults (four men, 16 women) ranging in age from 18 to 25 years (*M* = 20.7 years, *SD* = 1.8 years) and 20 older adults (four men, 16 women) ranging in age from 61 to 82 years (*M* = 71.9 years, *SD* = 5.5 years). The younger adults were university students and staff, the older participants were healthy community-dwelling volunteers. All participants were self-reported native speakers of American English, with no known history of stroke, Parkinson’s disease, or other neurologic involvement that might compromise their ability to perform the experimental task. Written informed consent was obtained from all participants according to a protocol approved by the Brandeis University Institutional Review Board.

All participants passed a visual acuity screen at or better than 20/50 using the Snellen eye chart at 20 feet (Hetherington, 1954) and the Jaeger close vision eye chart at 12 inches (Holladay, 2004). Audition was tested using a Grason-Stadler AudioStar Pro clinical audiometer (Grason-Stadler, Inc., Madison, WI, USA) *via* calibrated Eartone 3A insert earphones (E-A-R Auditory Systems, Aero Company, Indianapolis, IN, USA). The younger and older adults had a mean better-ear speech reception threshold (SRT) of 12.3 dB HL (*SD* = 3.8) and 22.5 dB HL (*SD* = 6.0), respectively. None of the participants reported being a regular user of hearing aids.

Both groups showed good verbal ability as indexed by the Shipley vocabulary test (Zachary, 1991). Older adults often show larger vocabulary scores than younger adults (Verhaeghen, 2003). In the present case the older adults showed a non-significant trend in this direction (younger adults *M* = 14.4; older adults *M* = 15.8), *t*_(37)_ = 1.85, *p* = 0.073 (A vocabulary score was unavailable for one younger adult).

#### Inhibitory Control

All participants were tested for inhibitory control ability using an arrow Flanker task (Eriksen and Eriksen, 1974; Verbruggen et al., 2004; Stins et al., 2007). In this test, participants were shown a group of five visual stimuli aligned horizontally on a computer screen. The center item was always an arrow, pointing either left or right. Participants were asked to indicate the direction of this center arrow using a left or right key-press. The surrounding items were either arrows facing the same direction as the center arrow (*congruent condition*), arrows facing the opposite direction (*incongruent condition*), or horizontal dashes (*neutral condition*). The incongruent condition is the condition of interest because it is here that effective inhibitory control is necessary for a correct response. As would be expected for a test of effectiveness of inhibitory control, the younger adults showed an accuracy advantage over the older adults [younger adults *M* = 94.1% (*SD* = 30.0); older adults *M* = 52.6% (*SD* = 36.0); *t*_(34)_ = 2.15, *p* = 0.039; scores for the Flanker task were missing for three younger adults and one older adult due to equipment failure].

### Stimuli

#### Visual Stimuli

The visual stimuli consisted of two words presented in written form horizontally on a computer screen. Printed words were used in this study rather than the pictures of objects to allow for a greater range of target words and distractors (e.g., Huettig and McQueen, 2007; McQueen and Viebahn, 2007; Salverda and Tanenhaus, 2010). The words were printed in upper case, with the right edge of the left word and the left edge of the right word approximately 15 cm from the edges of a 2.5 cm diameter central fixation point. One of the two words was always the last word of a spoken stimulus sentence (*target word*). The other word was either another word that might also be suggested by the sentence context (*semantic competitor*) or a word semantically unrelated to the sentence context (*unrelated word*). In all cases, the target and non-target words were selected to ensure that they did not share phonological onsets or ending rhymes as these can serve as an unintended source of competition (Allopenna et al., 1998; Ben-David et al., 2011; Farris-Trimble et al., 2014; Hadar et al., 2016).

#### Speech Stimuli

The stimuli consisted of 80 one- and two-syllable target words recorded by a native speaker of American English. To avoid an unintended influence of coarticulation cues each target word was recorded in the absence of a surrounding context, with computer speech editing used to splice each target word onto sentence frames that had been recorded by the same speaker. The sentence frames were 7- to 8-words in length with the final word missing, and were either suggestive of the sentence-final word, which in all cases was a common noun (e.g., “Some of the ashes dropped on the FLOOR”), or were uninformative as to the target word (“The word that they said was FLOOR”). This contrast was included to capture the effects of facilitation as well as competition.

Sentence frames and the sentence-final words were recorded with natural prosody and speech rate using Sound Studio v2.2.4 (Macromedia, Inc., San Francisco, CA, USA) that digitized (16-bit) at a sampling rate of 44.1 kHz. Root-mean-square (RMS) amplitude was equated across stimuli.

### Procedure

Participants were seated 60 cm from a 24^′′^ (1,920 × 1,080 pixel) computer screen with their head placed in a customized chin rest to stabilize head movement. At the start of each trial, participants used the computer mouse to place a cursor on a black central fixation point. Once the cursor was positioned, two words appeared on the computer screen, one on each side of the central fixation point.

Participants were allowed 2 s to familiarize themselves with the two words and their position on the computer screen. At the end of these 2 s, the central fixation point turned white and a stimulus sentence was presented binaurally over earphones (Eartone 3A insert earphones; E-A-R Auditory Systems, Aero Company, Indianapolis, IN, USA). Participants were told that the last word of each sentence (the target word) would always be one of the two words on the screen. Their task was to use the computer mouse to move the cursor from the central fixation point to the word that matched the sentence-final word and click on the mouse to confirm their selection.

Each participant heard a total of 80 test sentences, 20 trials in which the target word was paired with a word unrelated to the sentence context, and 20 in which the target word was paired with a semantic competitor (i.e., another word that might also fit the sentence context). A further 40 trials represented a no-context control condition, in which the sentence-final target word was preceded by the uninformative carrier phrase (“The word that they said was…”).

The experimental stimuli were counterbalanced across participants, such that, within each age group, by the end of the experiment each target word had been paired an equal number of times with a semantic competitor, an unrelated competitor, or with the uninformative sentence frame. Stimuli in each of the three conditions were inter-mixed in presentation.

#### Presentation Level

Presentation sound levels of the stimuli were individually adjusted for each participant to ensure the audibility of the stimulus sentences and sentence-final target words for both the younger and older adults. Participants were presented with low predictability sentences taken from the IEEE/Harvard sentence corpus (IEEE, 1969) presented initially at 10 dB above each participant’s better-ear SRT with the instruction to repeat each sentence as it was presented (e.g., “*The lake sparkled in the red, hot sun*.”). The sentences were recorded by the same speaker who recorded the experimental stimuli. The presentation levels were increased in 5 dB increments until the participant could repeat back two sentences with 100% accuracy. This sound level was used for that participant throughout the main experiment. This procedure resulted in sound levels for the main experiment between 15 and 30 dB HL for the younger adults (*M* = 23.3 dB HL) and between 30 and 50 dB HL (*M* = 37.8) for the older adults.

#### Eye-Gaze Data Acquisition

Throughout each trial the participant’s moment-to-moment eye-gaze position on the computer screen was recorded *via* a desk-mounted EyeLink 1000 Plus eye-tracking system (SR Research, Ontario, Canada), using a standard 9-point calibration procedure. The EyeLink acquired eye-position data at a rate of 1,000 Hz, recorded *via* MATLAB software (MathWorks, Natick, MA, USA). A drift correction was performed every 10 trials to account for natural drift over time.

Regions of interest (ROIs) defined an eye gaze on the center fixation point, a target word, or a non-target word. For the center fixation point, the boundary of the ROI was extended by 90 pixels from its edges. For the displayed words, the boundary of the ROI was extended by 300 pixels. This procedure avoided overlap between any ROIs (McMurray et al., 2017).

The proportion of time spent fixating on the target or distractor was calculated over 50 ms time bins, with each time bin calculated in a right-aligned moving window. Only data for correct trials were included in analyses. The main experiment was preceded by six practice trials using the same procedures as used in the experiment. None of these words or sentences was used in the main experiment.

## Results

### Gaze-Time on Target Words

**Figure 1:**
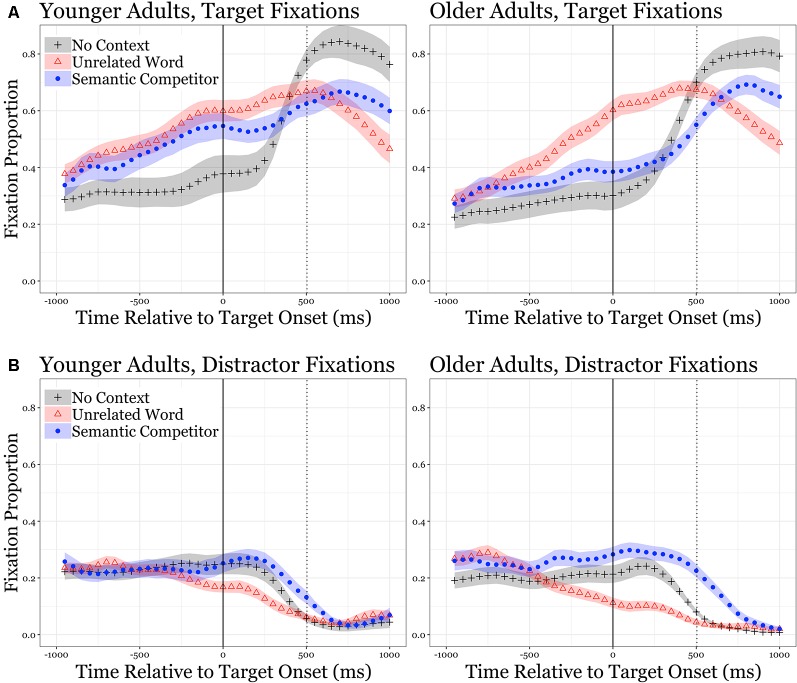
Eye fixations to target and distractors. The upper panels **(A)** show the proportion of eye fixation time on the target word for each condition for younger adults (left panel) and older adults (right panel). The lower panels **(B)** show the proportion of eye fixation time on non-target distractors for each condition for younger adults (left panel) and older adults (right panel). The zero-points on the abscissas mark the onset of the sentence-final word, with time to the left of this point representing the duration of the sentence frames. The dotted vertical lines indicate the average offset of the sentence-final words, with time to the right of this point representing the first 500 ms of a silent period that followed the offset of the sentence-final words. The shaded areas represent one standard error.

The upper left panel of Figure 1 shows the mean proportion of the time that the younger adults showed an eye fixation on the target word in each of the three conditions (semantic competitor, unrelated word, no context control) throughout an experimental trial. Data are shown beginning at 1,000 ms before the onset of a target word, representing on average the last three words of the sentence frame before the target word onset, through 500 ms following the end of the uttered target word. It can be seen from visual inspection of the left panel of Figure 1A that the proportion of time the younger adults’ eye-gaze fixed on the target word progressively increased as a constraining sentence unfolded in time, and even before the sentence-final target word was uttered. This influence of a linguistic context on the slope of the time-course of eye fixation on the target word can be seen for the younger adults to be similar whether the non-target word was a semantic competitor or a word unrelated to the sentence context.

A somewhat different pattern appears for the older adults as shown in the right panel of Figure 1A. Here one sees a steep increase in the time-course of gaze-time on the target word when an unrelated word was present, with this steepness indicative of older adults’ especially effective use of linguistic context in word recognition. When a semantic competitor was present, however, and in contrast with the younger adults, the increase in the older adults’ gaze time on the target word was sharply attenuated relative to when there was an unrelated distractor.

As would be expected for both age groups, when the target word was preceded by an uninformative carrier phrase in the No context control condition, there was a relatively flat-to-shallow gaze-time-on-target curve while hearing the carrier phrase. When the target word was uttered, gaze time on the target word showed a sharp increase, with this gaze time on the target word progressively increasing as the spoken target word unfolded in time.

#### Growth Curve Analysis

The eye fixation data for the target words shown in the two upper panels of Figure 1 were analyzed using growth curve analysis (Mirman, 2014), in which summed orthogonal polynomials were used to model changes in the overall pattern of the continuous variables over time (level, slope, inflection). This technique has its advantage in the sensitivity to potentially subtle effects while more comprehensively capturing the temporal dynamics of the eye fixations (Magnuson et al., 2007; Nozari et al., 2016).

The overall time course of target fixations was modeled with a third-order (cubic) orthogonal polynomial and with the condition (Semantic Competitor, Unrelated Distractor, No Context) and age group (younger adults, older adults) as fixed effects on all-time terms. For condition, the No Context condition was treated as the reference from which the relative parameters for Unrelated and Semantic Competitor distractor types were estimated, while for age group, younger adults were treated as the reference group and relative parameters were estimated for older adults.

For all growth curve analyses, the continuous variables were scaled and centered using the *scale* function. Each model was evaluated with participants and items as random effects as well as random slope terms for all polynomials and variables tested (Barr et al., 2013). The fixed effects were added into the model in the order listed for each separate analysis with each respective interaction entered after the main effects, starting with polynomial interactions followed by fixed-effect interactions in order. The effects on model fit were evaluated using model comparisons of the change in log-likelihood using the analysis of variance (ANOVA) function (Mirman, 2014), beginning with a base model containing only the first and second polynomials, then adding the third polynomial, and finally the fixed effects and interactions in order. All analyses were carried out in R version 3.5.2 using the *lme4* package (version 1.1–19) and the function *lmer* to fit the models. Gaze data was analyzed from 1,000 ms before to 1,000 ms after the target word onset.

The results of the analysis are shown in Table 1, where it can be seen that there was a significant effect of condition on the intercept and all polynomial terms. The interaction between condition and age group on the intercept was also significant, as was the interaction effect of condition and age group on the slope term and the cubic term. This is consistent with the differential slowing for older adults when there was a semantic competitor relative to when there was an unrelated distractor.

**Table 1:** Growth curve analysis of target fixations.

Fixed effects	Estimate (SE)			*χ*^2^	*df*	*p*-value
	Unrelated		Sem comp			
Intercept	0.10 (0.04)		
Linear (slope)	−0.40 (0.04)		
Quadratic	0.25 (0.06)		
Cubic	0.64 (0.13)			24.10	1	<0.001***
Condition (intercept)	0.07 (0.01)		−0.02 (0.01)	35.86	2	<0.001***
Condition (slope)	−0.14 (0.02)		0.05 (0.02)	46.62	2	<0.001***
Condition (quad.)	−0.30 (0.02)		−0.01 (0.01)	203.77	2	<0.001***
Condition (cubic)	−0.55 (0.02)		0.01 (0.02)	649.35	2	<0.001***
Age Group (int.)	0.06 (0.04)			1.93	1	0.165
Age Group (slope)	0.01 (0.04)			0.20	1	0.654
Age Group (quad.)	−0.05 (0.06)			0.59	1	0.441
Age Group (cubic)	−0.10 (0.13)			0.41	1	0.520
Cond. × Age Gr. (int.)	−0.01 (<0.01)		0.02 (<0.01)	45.99	2	<0.001***
Cond. × Age Gr. (slope)	0.06 (0.02)		−0.05 (0.01)	16.73	2	<0.001***
Cond. × Age Gr. (quad.)	0.01 (0.02)		−0.02 (0.01)	1.65	2	0.438
Cond. × Age Gr. (cubic)	0.01 (0.02)		−0.04 (0.01)	7.42	2	0.024*

Notes. *p < 0.05, ***p < 0.001.

### Gaze-Time on Distractor Words

The two lower panels of Figure 1B show mean gaze-times on the non-target words for each of the three conditions and reflect a progressive decline in gaze-times on non-target words as the gaze times on the target words (upper panels of Figure 1) increased. The results of a growth curve analysis for these data are given in Table 2, where it can be seen that there was a significant effect of condition on the intercept and all polynomial terms. The interaction between condition and age group on the intercept also was significant, as well as the interaction effect of condition and age group on the slope term. This reflects the differential effect on the older adults’ distractor gaze pattern when a semantic competitor was present, such that older adults looked at the on-screen distractor differentially more than the younger adults when it was a semantic competitor.

**Table 2:** Growth curve analysis of distractor fixations.

Fixed Effects	Estimate (SE)			*χ*^2^	*df*	*p*-value
	Unrelated		Sem Comp
Intercept	−0.08 (0.03)		
Linear (slope)	0.10 (0.04)		
Quadratic	−0.26 (0.03)		
Cubic	−0.56 (0.06)			49.33	1	<0.001***
Condition (intercept)	−0.02 (0.01)		0.05 (0.01)	26.23	2	<0.001***
Condition (slope)	0.20 (0.02)		−0.10 (0.01)	128.40	2	<0.001***
Condition (quad.)	0.07 (0.02)		−0.02 (0.01)	12.76	2	<0.001***
Condition (cubic)	0.04 (0.02)		−0.06 (0.01)	19.20	2	<0.001***
Age Group (int.)	0.00 (0.03)^†^			0.11	1	0.742
Age Group (slope)	0.00 (0.04)			0.04	1	0.835
Age Group (quad.)	0.03 (0.03)			0.91	1	0.339
Age Group (cubic)	0.04 (0.05)			0.39	1	0.534
Cond. × Age Gr. (int.)	0.00 (<0.01)		−0.02 (<0.01)	73, 92	2	<0.001***
Cond. × Age Gr. (slope)	−0.06 (0.02)		0.03 (0.01)	9.77	2	0.008**
Cond. × Age Gr. (quad.)	0.01 (0.02)		0.02 (0.01)	2.20	2	0.332
Cond. × Age Gr. (cubic)	0.01 (0.02)		0.02 (0.01)	1.57	2	0.456

Notes. **p < 0.01, ***p < 0.001. ^†^0.00 indicates a value between 0 and 0.01; −0.00 indicates a value between −0.01 and 0.

### Inhibitory Control

**Figure 2:**
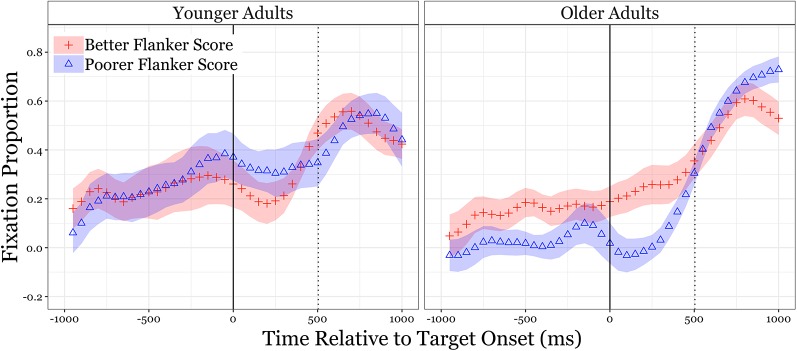
Eye fixations and inhibitory control ability for younger and older adults in the semantic competitor condition. Fixations are shown as a subtraction, with semantic competitor fixations subtracted from the target fixations. The zero-point on the abscissas mark the onset of the sentence-final word, with time to the left of this point the duration of the sentence frames. The dotted vertical lines indicate the average offset of the sentence-final words, with time to the right of this point showing the first 500 ms of a silent period that followed the offset of the sentence-final words. The shaded areas represent one standard error.

Although the Flanker inhibitory control measure is treated as a continuous variable in the analysis that follows, its contrasting effects can be illustrated in Figure 2 which shows the pattern of eye-fixations in the Semantic Competitor condition for participants with better vs. lower inhibition scores based on a median split of Flanker accuracy within each age group (Younger adults: better *M* = 100%; lower *M* = 55.6%. Older adults: better *M* = 82.5%; lower *M* = 19.4%). To illustrate this contrast fixations in Figure 2 are shown as a subtraction, with Semantic Competitor fixations subtracted from Target fixations.

In contrast to the young adults (left panel), who show considerable overlap in the eye-fixation time curves for those with better and poorer Flanker scores, it can be seen that the older adults (right panel) with poorer Flanker scores spent less gaze-time on the target word than those with better Flanker scores. This difference is especially notable before the target word onset and for just under half of the average target word duration. Once beyond this point, the eye-fixation curves of the older adults with poorer Flanker scores start to converge with those with better Flanker scores (for ease of presentation the eye fixation data in Figure 2 are shown as a subtraction, with fixations on the Semantic Competitor subtracted from Target fixations).

#### Growth Curve Analysis

Table 3 shows the result of a growth curve analysis of eye-gaze times with Flanker score treated as a continuous variable. This analysis focused on the Semantic Competitor condition with the analysis performed on Target fixation times minus Semantic Competitor fixations across time. Fixations were again modeled with a third-order (cubic) orthogonal polynomial and fixed effects of Flanker score (continuous variable) and age group (younger adults, older adults) on all-time terms, and a maximal random effects structure. For the age group, younger adults were treated as the reference group and relative parameters were estimated for older adults. All continuous variables were scaled and centered using the *scale* function. It can be seen in Table 3 that Flanker scores had a significant effect on the slope term, such that better inhibitory control ability predicted a steeper slope.

**Table 3:** Growth curve analysis for Flanker scores in the semantic competitor condition.

Fixed Effects	Estimate (SE)	*χ*^2^	*df*	*p*-value
Intercept	0.03 (0.03)			
Linear (slope)	−0.29 (0.04)			
Quadratic	0.01 (0.04)			
Cubic	0.48 (0.07)	30.56	1	<0.001***
Flanker (intercept)	−0.00 (0.02)^†^	1.34	1	0.247
Flanker (slope)	0.11 (0.04)	7.20	1	0.007**
Flanker (quad.)	−0.05 (0.04)	1.76	1	0.185
Flanker (cubic)	−0.07 (0.07)	2.40	1	0.122
Age Group (int.)	−0.07 (0.02)	7.24	1	0.007**
Age Group (slope)	−0.01 (0.04)	0.03	1	0.867
Age Group (quad.)	−0.00 (0.04)	0.01	1	0.910
Age Group (cubic)	0.08 (0.07)	1.16	1	0.281
Flanker × Age Gr. (int.)	0.05 (0.02)	4.74	1	0.029*
Flanker × Age Gr. (slope)	0.00 (0.04)	0.01	1	0.934
Flanker × Age Gr. (quad.)	−0.05 (0.04)	1.49	1	0.223
Flanker × Age Gr. (cubic)	−0.05 (0.07)	0.42	1	0.516

Notes. *p < 0.01, **p < 0.01, ***p < 0.001. ^†^0.00 indicates a value between 0 and 0.01; −0.00 indicates a value between −0.01 and 0.

As might be expected, it can be seen in Table 3 that age group had a significant effect on the intercept, indicating that older adults had an overall smaller difference between their Target and Semantic Competitor fixations in this condition. Flanker score also significantly interacted with age group on the intercept, indicating that there was a larger effect of inhibitory control ability on the overall difference between Target and Distractor fixations in the older adult group.

### Accuracy of the Overt Response

As indicated, the analyses of eye-gaze data were conducted for just those cases where the participant’s initial mouse click was on the correct target word. The younger and older adults showed a similar pattern of initial response accuracy. The younger adults” mean initial response accuracy was 92.1% (*SD* = 16.8) for the No Context condition, 96.0% (*SD* = 5.4) for the Unrelated word condition, and 75.3% (*SD* = 21.1) for the Semantic Competitor condition. The older adults mean initial response accuracy was 89.5% (*SD* = 17.0) for the No Context condition, 93.3% (*SD* = 10.9) for the Unrelated word condition, and 76.5% (*SD* = 19.7) for the Semantic competitor condition. These data were submitted to a 3 (Condition: unrelated word, No context, Semantic competitor) × 2 (Age group: younger, Older) mixed-design ANOVA with conditions as a within-participants variable. The ANOVA confirmed a significant main effect of condition, *F*_(2,38)_ = 26.78, *p* < 0.001, $\eta_{\text{p}}^{2}$ = 0.41, but no main effect of age, *F*_(1,38)_ = 0.11, *p* = 0.741, $\eta_{\text{p}}^{2}$ = 0.003. The similarity of the accuracy pattern across conditions for the two age groups was reflected in the absence of a significant Condition x Age group interaction, *F*_(2,38)_ = 0.34, *p* = 0.714, $\eta_{\text{p}}^{2}$ = 0.68.

Bonferroni-corrected pairwise comparisons were conducted to clarify the main effect of the condition. The data for the two age groups were combined as there was no main effect of age. The Bonferroni tests confirmed that the main effect of conditions was due to participants being significantly less accurate when a Semantic Competitor was present compared to when an Unrelated word was present (*p* < 0.001), and less accurate with the presence of a Semantic Competitor compared to the No Context control (*p* < 0.001). Accuracy for the Unrelated word condition did not differ significantly from accuracy for the No Context control (*p* = 0.205). Participants at times corrected an initial incorrect response, particularly in the Semantic Competitor condition, where the younger adults’ mean eventual-response accuracy was 87.8% (*SD* = 19.5), and the older adults mean eventual-response accuracy was 87.4% (*SD* = 21.6).

## Discussion

The present results add to extant knowledge in several ways. First, the use of eye-tracking demonstrates a facilitation effect of linguistic context on the speed of word recognition operating on-line as speech is being heard. That is, we observed a progressive increase in participants’ gaze-time on a contextually constrained target word that began to appear even before the sentence-final target word was heard (for supporting data see Ayasse et al., 2017). This would be consistent with a dynamic system in which the progressive unfolding of a context sentence results in a corresponding increase in the activation level of the lexical representation of the sentence-final target word from its pre-context resting state.

As distinct from response bias, or so-called modular model of word recognition, in which context comes into play only after the mental representation of the word has been activated (e.g., Swinney, 1979; Forster, 1981), we thus see as the mechanism underlying the rapid recognition of words in context as consistent with *a priori* activation and on-line interactive models of word recognition (e.g., Morton, 1969; Marslen-Wilson and Zwitserlood, 1989; Sereno et al., 2003). To the extent this is the case, this effect of context was seen to hold for the older as well as the younger adults (Cohen and Faulkner, 1983; Wingfield et al., 1991, 1994; Pichora-Fuller et al., 1995; Lash et al., 2013).

Our major interests, however, were first to test the postulate of an age-related inhibition deficit affecting spoken word recognition, and second, to determine the locus of this deficit. It was seen that the younger adults showed a somewhat similar slope of their eye-gaze on the target word growth curves, regardless of the presence or absence of a semantic competitor in the response set. In contrast, the older adults showed a shallower growth curve when the non-target word was a semantic competitor that might also fit the sentence context.

This contrasting effect of the presence of semantic competitor on eye-fixation times is what one would expect if older adults were less able than young adults to inhibit interference, in this case, from the presence of a semantic competitor. That is, the postulated age-related inhibition deficit characterized by Hasher and Zacks (1988) and Lustig et al. (2007) that has been shown to interfere with successful speech recognition at the off-line level (Sommers, 1996; Sommers and Danielson, 1999; Taler et al., 2010; Lash et al., 2013; Lash and Wingfield, 2014; Dey and Sommers, 2015) is now shown to also retard the speed of word recognition at the level of on-line processing.

Use of the Flanker task as an independent index of inhibitory control (Verbruggen et al., 2004; Stins et al., 2007) shed additional light on an age-sensitive inhibition deficit as a predictor of word recognition speed. In the critical condition, in which a semantic competitor was present on the computer screen along with the target word, those older adults with poorer Flanker scores spent less gaze-time on the target word (and more time on the semantic competitor) than those with better Flanker scores. That is, the differential effect of interference in the older relative to the younger adults’ on-line recognition speed was driven at least in part by differences in inhibitory control ability.

These data thus illustrate a negative side to hearing a word within a sentence context, in which interference from other words that might also fit the sentence context appears to retard the speed of word recognition. They further show this effect of interference to be differentially greater for older adults, with this effect traceable to differences in general inhibition ability as indexed by Flanker scores. This is consistent with work by Marrufo-Pérez et al. (2019), who found in an off-line study that word recognition was poorer for words positioned later in a sentence and that this effect was explained by inaccurate early predictions that were not fully inhibited (see also Lash and Wingfield, 2014). It is also worth noting that there is some evidence in prior literature that the benefit of context is greater for older adults, and particularly older adults with a mild-to-moderate hearing impairment, than younger adults (e.g., Signoret and Rudner, 2019), although some studies have also found the benefit to be approximately equal (see Cohen and Faulkner, 1983; Wingfield et al., 1991; Pichora-Fuller et al., 1995; Dubno et al., 2000; Benichov et al., 2012).

Interestingly, this differential age effect did not appear in the accuracy of participants’ overt response selections, suggesting that, at least for this paradigm, the initial age difference had dissipated by the time participants made their behavioral response selection. It is possible that, in contrast to the rapidity of saccadic eye-movements, making an overt response selection may have obscured subtle age effects due to uncontrolled speed-accuracy tradeoffs that may have varied from trial-to-trial as well as between participants.

Two additional caveats should be noted. At the empirical level, the present results focused on the domain of context effects on spoken word recognition. They do not necessarily imply a domain-general inhibition deficit that applies across all cognitive domains and time scales. This is a question that is beyond the scope of the present study (see Burke and College, 1997; Zacks and Hasher, 1997; Burke and Osborne, 2007). At the theoretical level, in invoking the notion of an age-related inhibition deficit it should be acknowledged that conceptions of, and hence contrast between, executive function, inhibitory control, working memory, and their relations, are not as yet fully formed in the cognitive literature (see Miyake et al., 2000; Engle, 2002; Cowan, 2005; McCabe et al., 2010; Wilhelm et al., 2013; see the review in Wingfield, 2016).

### Conclusions

Within these caveats, the present data illustrate the two sides to the effects of the linguistic context. On the positive side, a constraining linguistic context facilitates the ease of word recognition. On the negative side, potential interference from other words that might also fit the sentence context can slow word recognition, and particularly so for older adults, with both effects appearing on-line as the stimuli were being heard. The ease of word recognition can thus be seen as a balance between contextual facilitation and interference effects, both of which operate when younger and older adults hear words spoken within a linguistic context. The age difference appears only in the ratio of these two effects as they contribute to this balance.

## Data Availability Statement

The datasets generated for this study are available to any qualified researcher upon request to the authors.

## Ethics Statement

The studies involving human participants were reviewed and approved by Brandeis University Institutional Review Board (IRB). The patients/participants provided their written informed consent to participate in this study.

## Author Contributions

NA and AW collaborated on the experimental design, data analysis, data interpretation, and drafting of this manuscript.

## Conflict of Interest

The authors declare that the research was conducted in the absence of any commercial or financial relationships that could be construed as a potential conflict of interest.
